# The cause analysis of benign uretero-ileal anastomotic stricture after radical cystectomy and urinary diversion

**DOI:** 10.3389/fonc.2022.1070141

**Published:** 2022-12-13

**Authors:** Zhenghong Liu, Bin Zheng, Yuqi Hu, Haichang Li, Xiaowen Qin, Xuanhan Hu, Shuai Wang, Heng Wang, Pu Zhang, Qijun Wo, Li Sun, Yixuan Mou, Feng Liu, Jianxin Cui, Dahong Zhang

**Affiliations:** ^1^ Urology and Nephrology Center, Department of Urology, Zhejiang Provincial People’s Hospital, Affiliated People’s Hospital, Hangzhou Medical College, Hangzhou, China; ^2^ Department of Organ Transplantation, The Affiliated Yantai Yuhuangding Hospital of Qingdao University, Yantai, China

**Keywords:** bladder cancer, urinary diversion, anastomosis, radical cystectomy, uretero-ileal anastomotic stricture

## Abstract

**Background:**

Benign uretero-ileal anastomotic stricture (UIAS) is a major complication following radical cystectomy (RC) and ileal orthotopic bladder substitution, and it can occur in combination with other complications. But risk factors for patients with UIAS have not been well described.

**Material and methods:**

We retrospectively reviewed 198 patients treated with RC for bladder cancer from 2014 to 2019 at the Zhejiang Provincial People’s Hospital. Patient demographic and clinical variables were examined to determine the risk factors associated with UIAS by univariate and multivariate logistic regression analysis.

**Results:**

A total of 180 patients into the group standards and in all 360 uretero-ileal anastomoses. Among the above cases, 22 patients developed UIAS, including 10 cases of left UIAS, nine cases of right UIAS, and three cases of bilateral UIAS. There was no difference in demographic, operative, or perioperative variables between patients with and without UIAS. In a multivariate analysis, after adjusting for gender, age, surgical methods, and underlying diseases, intraoperative or postoperative blood transfusion (HR = 0.144, P <0.01), postoperative urinary tract infection (HR = 3.624, P <0.01), and extracorporeal bladder anastomosis (HR = 3.395, P = 0.02) significantly increased the risk of UIAS.

**Conclusions:**

In our experience, intraoperative or postoperative blood transfusion, postoperative urinary tract infection, and extracorporeal neobladder anastomoses increased the risk of UIAS after radical cystectomy and ileal orthotopic bladder substitution surgery. Further studies with larger samples are necessary to validate this result.

## Introduction

Bladder cancer was the ninth most common cancer worldwide and the 13th most common cause of death, according to the latest research data. In recent years, the incidence of bladder cancer has been increasing year by year (1). The main performance is male incidence rate is higher than female, rural incidence rate is higher than city (1). Diagnosis, treatment, and 5-year survival rates for bladder cancer have remained largely unchanged since the 1990s. Radical cystectomy (RC) with urinary diversion (UD) is standard therapy for muscle-invasive bladder cancer and high-risk non-muscle-invasive disease (2). Most strictures occur between 6 months and 18 months after surgery. The overall complication rate is reported to be as high as 25%–35% (3). The published rate of UIAS after urinary diversion has a varying incidence in the literature (4). The incidence of postoperative benign UIAS has been reported to be 1% to 30% and varies considerably. Besides, complications after ileal bladder surgery are mainly urinary tract infection, incision infection, uretero-ileum stricture, intestinal fistula, intestinal obstruction, etc. (5, 6). Among them, urinary tract infections are the most common. If the ureteral outlet stricture is not treated in time, it is prone to complicated upper urinary tract infections, stones, renal insufficiency, and other serious complications. It has been proposed that the cause of UIAS is likely multifactorial. The causes of UIAS include anastomotic fibrosis, inflammation, and tumor recurrence, among which fibrosis is the most common factor (7). Excessive dissection and freeing of the ureter may lead to ureteral damage and ischemia, followed by inflammation, fibrosis, and scar formation (7). We retrospectively analyzed our surgical experience and demographic data in order to determine the risk factors for the formation of UIAS.

## Materials and methods

We obtained approval from our institutional review board before initiating this analysis. A retrospective database of all patients who underwent RC with an ileal conduit or an ileal orthotopic neobladder for bladder cancer at Zhejiang Provincial People’s Hospital from 2014 to 2019 was analyzed. Patients’ clinical characteristics were retrieved from hospital archives, including gender, age, body mass index, the American Society of Anesthesiologists (ASA), comorbidities, chemotherapy history, drinking history, and smoking history. Preoperative data: hemoglobin, WBC level, creatinine, albumin, uric acid level, alanine transferase, aspartate transferase, etc. Intraoperative data included intraoperative blood transfusion, lymph node dissection, the duration of the operation, and the preparation of a new bladder. Postoperative: hemoglobin, creatinine, postoperative urinary tract infection, average length of hospital stay. The diagnosis of UIAS is mainly made through radiography, enhanced CT, and three-dimensional reconstruction of the urinary system. If there is clear radiologic evidence, UIAS has occurred. Malignant strictures were excluded from the study. An enhanced CT scan of the urinary system would be performed every 3 months within 1 year after surgery, every 6 months within 1–5 years, and annually after 5 years. The shortest observation period is 1 year.

All the risk variables with UIAS were analyzed as a binary variable and assessed by univariable logistic regression analysis to detect outcome variables with significant odds ratios.

The variables that attained significance in univariate analysis included intraoperative or postoperative blood transfusion, extracorporeal neobladder anastomoses, and postoperative urinary tract infection. They were entered into a multivariable logistic model with an interaction term to ascertain whether they retained significance. All statistical tests were implemented at p ≤0.05 significance level.

## Results

A total of 198 patients with radical bladder cancer were included in the study. Cases, which occur Malignant UIAS, with other Malignant tumor, loss to follow-up nine patients, finally only 180 patients into the group standards. The average postoperative hospital stay was 14.4 days. Every UIAS is a unit at risk of developing stricture and in all 360 uretero-ileal anastomosis, stricture patients a total of 22 cases, 10 cases were on the left side of the narrow, the right side of the narrow nine cases, bilateral stricture (three cases), stricture rate was 6.9%. Demographic information and clinical data collected are shown in **Tables 1**, **2**.

**Table 1:** Summary of continuous variables collected.

variables	Minimum	Median	Maximum
**Age**	43	67	92
**BMI**	16	22	28
**ASA score**	1	2	3

**Table 2:** Summary of categorical variables recorded.

Variables	N
Age	
43–67	93
67–92	87
BMI	
Normal	110
Abnormal	70
ASA score	
**1**	116
**2**	41
**3**	23
Sex	
Male	159
Female	21
Diabetic	
Yes	154
No	26
Smoking	
Yes	102
No	78
Drinking	
Yes	50
No	130
Preoperative HB	
≥120 g/L	155
<120 g/L	25
Preoperative WBC	
>10 × 109/L	13
≤10 × 109/L	167
Preoperative albumin	
≥40 g/L	106
<40 g/L	74
Preoperative Uric acid level	
>357 umol/L	56
≤357 umol/L	124
Preoperative Urea level	
>8.8 mmol/L	39
≤8.8 mmol/L	141
Preoperative Creatinine	
>123 umol/L	20
≤123 umol/L	160
Preoperative ALT	
>40 U/L	18
≤40 U/L	162
Preoperative AST	
>35 U/L	15
≤35 U/L	165
Preoperative cruenturesis	
Yes	173
No	7
Urine microscopy for white blood cells	
Yes	119
No	61
Blood transfusion	
Yes	22
No	158
Bricker anastomotic technique	
Yes	101
No	79
Intraoperative bleeding>400 ml	
Yes	79
No	101
Running Anastomosis	
Yes	145
No	35
Operation time>4 h	
Yes	141
No	39
Robot assisted surgery	
Yes	105
No	75
Intracorporeal neobladder anastomoses	
Yes	120
No	60
Postoperative HB (g/L)	
≥120 g/L	109
<120 g/L	71
Postoperative WBC	
>10 × 109/L	108
≤10 × 109/L	72
Postoperative Creatinine	
>123 umol/L	20
≤123 umol/L	160
Postoperative urinary tract infection	
Yes	40
No	140

**Figure 1:**
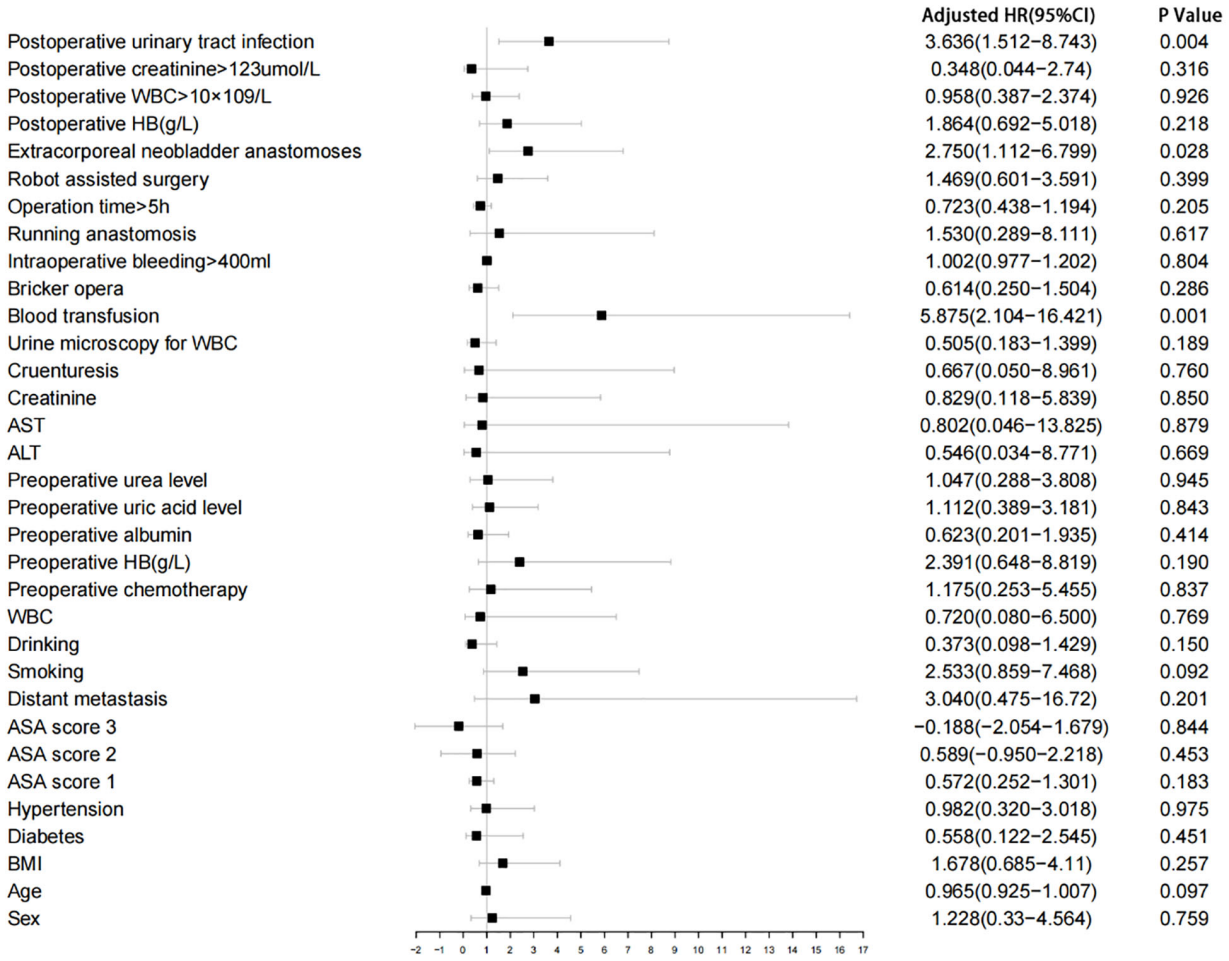
Univariate logistic regression analysis. WBC, white blood cell; ALT, Alanine transferase; AST, Aspartate transferase; HB, Hemoglobin.

Of 180 patients, the median age was 67 years, with 159 (88.3%) males. A total of 60 (33.3%) cases were occurred extracorporeal neobladder anastomoses, 22 (12.2%) patients were undergone intraoperative or postoperative blood transfusion and 40 (22.2%) patients were developed urinary tract infection. The incidence of bilateral strictures was 13.6%. Left side stricture was 45.5%, while the right side was 40.9%. The median interval from postoperative to diagnosis of stricture was 11 months. Univariate logistic regression analysis with narrow correlations between each variable is summarized in **Figure 1**.

Multivariate Logistic regression analysis was performed on UIAS risk factors with statistical differences (P <0.05) in univariate analysis. Intraoperative or postoperative blood transfusion (HR = 0.144, P <0.01), postoperative urinary tract infection (HR = 3.624, P <0.01), and extracorporeal bladder anastomosis (HR = 3.395, P = 0.02) were the variables most strongly associated with UIAS. Besides, the results of three variables that were taken into a multivariable logistic regression analysis are shown in **Table 3**.

**Table 3:** Multivariable Logistic regression analysis.

Multivariate analysis	HR	P
Blood transfusion		
No	1	
Yes	0.144 (0.046–0.451)	0.001
Bladder anastomosis		
Intra	1	
extra	3.395 (1.241–9.283)	0.017
Urinary tract infection		
No	1	
Yes	3.624 (1.41–9.31)	0.007

**Figure 2:**
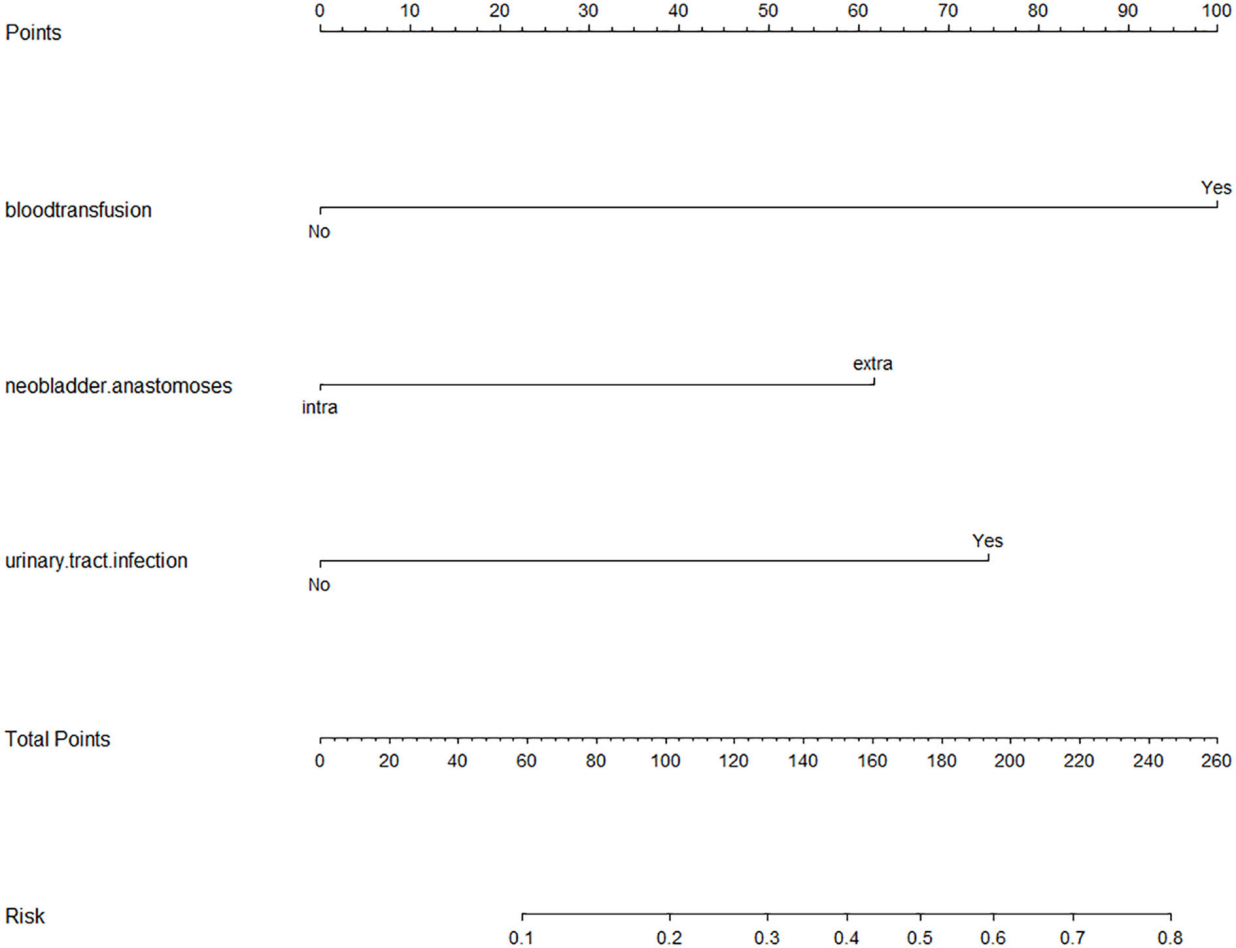
Nomogram based on Logistic regression model. Abbreviations: HR, hazard ratio. The scores of the three risk factors are added together to form the total score, ranging from 0 to 260 points. The percentage corresponding to the total score downward is the probability of UIAS in patients with Radical cystectomy with urinary diversion.

Nomogram based on a logistic regression model: Based on the R software, assign values to the inclusion indicators of patients with UIAS and use the regression coefficient values corresponding to the three statistically significant indicators in the logistic regression analysis and the R language software program to obtain the score corresponding to each indicator, and then draw a list of items predicting the occurrence of bacterial infections. In the nomogram, sum the scores corresponding to each indicator and find the corresponding UISA probability through the total score. The results are shown in **Figure 2**.

In the model fitted this time, the likelihood ratio test result of whether all parameters are 0 is P <0.05, that is, the OR value of at least one variable among the included variables is statistically significant, and the model is generally meaningful. The P-value of the goodness of fit is not less than the test level (p >0.05), the information in the current data has been fully extracted, and the goodness of fit of the model is high. This study found that anastomosing a new bladder in the extracorporeal setting compared to anastomosing a new bladder in the intracorporeal setting has an increased risk of UIAS. Blood transfusions during or after surgery also increased the risk of UIAS. At the same time, consistent with other related research, postoperative urinary tract infection was also associated with an increased risk of UIAS. As the risk factors of patients increase, the proportion of UISA has a strong tendency to increase. At least one risk factor has a strong predictive effect on the development of UIAS.

## Discussion

RC and standard pelvic lymph node dissection: Bilateral pelvic lymph nodes were exposed and dissected. Free ureter from the ureteral wall segment. The peritoneum and vascular sheath were opened, and the internal and external iliac and obturator lymph nodes were dissected. Free cystorectal space and anterior bladder space. The bladder and prostatic ligaments were dealt with. Treatment of the prostate and urethra: the prostate and bladder are completely removed. Preparation of a new bladder: A mesangial enteral loop was taken about 20–30 cm from 10 to 20 cm away from the ileocecal part, and the broken ends of the proximal and distal ileocecal parts were anastomosed end to end or side to side, and the mesangial was sutured.

Furthermore, the following points should receive special attention in the operation: 1. minimize free tissue around the ureter to reduce blood supply; 2. avoid clamping the broken end of the ureter; 3. Ureteral length is retained to avoid excessive ureteral tension; and 4. ureteral stents are implanted to prevent stenosis.

Several retrospective studies have been conducted to identify risk factors associated with UIAS after radical cystectomy and ileal conduit formation. Current studies suggest that postoperative anastomotic leakage, postoperative urinary tract infection, and anastomotic ischemia would increase UIAS (8). In our study, intraoperative blood loss was not well reflected due to the subjective evaluation of intraoperative bleeding, intraoperative and postoperative blood transfusion patients had greater intraoperative bleeding, which may lead to anastomotic ischemia and cause anastomotic stricture. Postoperative urinary tract infection also increased the risk of anastomotic stricture, which may be related to delayed wound healing and scar hyperplasia, consistent with previous domestic and foreign research results.

Of course, it was important to pay proper attention to delicate surgical techniques. Careful management of the broken ureter end, degree of tissue dissociation, interruption time of the ureteral blood supply, and tension after ureteral replantation were considered during the operation. It remains cannot be accurately quantified and measured accurately.

There have been many previous studies trying to find increased risk factors for UIAS, but the exact cause is still unclear. Hoag et al. found in their research on risk factors for UIAS that diabetes and elevated levels increase the UIAS rate. It may be due to microvascular disease that the distal ureter becomes sensitive (9). In addition, age, body mass index, hemoglobin level, and ASA were not predictive factors for UIAS formation, which was consistent with our results.

Large et al. suggested that the running anastomosis and postoperative urinary tract infection might be related to UIAS. He believed that interrupted anastomosis had less effect on the blood supply at the anastomotic site, therefore the stricture rate was lower (10). In our study, there was no significant difference in the stricture rate between interrupted anastomosis and running anastomosis. Studies in our center currently suggest that running anastomoses have no significant effect on blood supply at the uretero-ileal anastomosis. Meanwhile, the influence of postoperative urinary tract infection on the stenosis rate was consistent with the study.

In previous reports on the prediction model of the incidence of radiotherapy and UIAS, it was identified that irradiated tissue has abnormal maturation of fibroblasts, leading to delayed healing, fibrosis, and scar formation in the distal ureter, as well as radiation-induced endarteritis that increased the risk of UIA ischemia. However, Katkoori et al. evaluated previous pelvic radiation for UIAS in 526 patients who had RC and UD between 1992 and 2008. They suggested that there was no significant difference between those with previous pelvic radiotherapy (pRT) and those without previous pRT (1.5% vs. 1.6%, P = 0.6) (3).

The conclusions on the influence of different surgical methods on the incidence of UIAS were different. Davis et al. found that there was no significant difference in the incidence of UIAS for Bricker and Wallace anastomoses (11). On the contrary, Kouba et al. found that Wallace anastomoses had a lower risk than Bricker anastomoses, but did not rule out a BMI effect on the results (12). In our study, there was no significant difference in the incidence of stenosis between Wallace and Bricker, meanwhile, and BMI had no significant effect on UIAS.

In the Mullins study, for the 192 patients with radical resection of the bladder and the UD retrospective study, it was found that in patients with the Wallace and Bricker operation method, stenting of the UIAS for the postoperative stricture rate has no obvious influence but is decreased after the incidence of intestinal obstruction (13). In our model, all patients underwent postoperative ureteral stent implantation to help control the formation of UIAS by transforming risk factors, and the effect of UIAS could not be determined. Current clinical experience shows that ureteral stents do not increase the rate of strictures. In the current study, the left UIAS was more marked than the right UIAS, as the left ureter passed beneath the sigmoid mesentery, increasing mobilization and tunneling under the sigmoid colon. However, in our model, although the left stricture rate was higher than the right stricture, there was no statistically significant difference (p = 0.54).

Anderson et al. in their study found that there was no significant difference in the rate of stricture between the robot-assisted and open groups (12.6% vs. 8.5%, p = 0.21) (14). In our institution, all undergoing radical cystectomy and ileal orthotopic bladder substitution patients adopt laparoscopic and robot assisted surgery and no significant difference in robot-assisted group and laparoscopic group.

Richards et al. found the median time to stricture formation on the right and left ureters to be 235 and 232 days, respectively. Besides, the length of the distal ureter resected did not significantly influence the stricture rate. The reason for the benign UIAS following was multifactorial (7).

There were several possible explanations for the finding of extracorporeal neobladder anastomoses as a risk factor for modified UIAS. The free length of the ureter in the external bladder anastomosis is 5–10 cm longer than the free length of the ureter in the internal bladder anastomosis, and the blood supply of the ureter is more affected. Besides, experienced surgeons choose a larger proportion of the intracorporeal neobladder anastomoses, better protection of the ureteral blood supply, more delicate tissue processing, more detailed surgical skills, less probability of uretero-ileum anastomosis leakage, and therefore a lower rate of stricture.

Nevertheless, this study had some limitations that are important to mention. For the retrospective studies and due to many confounding factors of collected data, retrospective studies often have substantial advantages to biomarker evaluation. The cases included in this study are all from the Zhejiang Provincial People’s Hospital, which may be biased in the establishment of risk prediction models. Future research can increase the sample size or multi-center data to reduce the bias in experiment inclusion and improve the accuracy of risk assessment model predictions. Since some patients may have stricture but have no symptoms, it is still unknown whether the rate of stricture was not detected in time.

## Conclusions

Benign UIAS after ileal UD had a multifactorial etiology. In our series, intraoperative or postoperative blood transfusion, postoperative urinary tract infection, and extracorporeal neobladder anastomoses increased the risk of UIAS after radical cystectomy and ileal ureteral diversion. In addition, reducing the dissociation of the distal end of the ureter and protecting the blood supply of the ureter are essential to reducing the rate of stricture.

## Data Availability

The original contributions presented in the study are included in the article/supplementary material. Further inquiries can be directed to the corresponding authors.
